# DNA aptamers from whole-serum SELEX as new diagnostic agents against gastric cancer

**DOI:** 10.1039/c8ra08642g

**Published:** 2019-01-09

**Authors:** Yue Zheng, Yunwang Zhao, Ya Di, Chenlin Xiu, Lei He, Shiqi Liao, Dongdong Li, Baihai Huang

**Affiliations:** The First Hospital of Qinhuangdao Qinhuangdao 066000 China zhengyueqhd@sina.com +86-0335-590-8121; College of Life Sciences, Lanzhou University Lanzhou 730000 China; Central Pharmaceutical Research Institute, Shijiazhuang Pharmaceutical Group Shijiazhuang 050041 China; College of Environment &Chemical Engineering, Yanshan University Qinhuangdao 066004 China

## Abstract

Gastric cancer is still among the leading causes of cancer deaths worldwide. Despite the improvements in diagnostic methods, the status of early detection has not been achieved so far. Early diagnosis of gastric cancer may significantly improve the cure rate of patients. Therefore, a new diagnostic method is needed. In this study, subtractive SELEX was performed to screen gastric cancer serum-specific DNA aptamers by using gastric cancer serum and normal serum as the target and negative serum, respectively. Four highly specific aptamers generated for gastric cancer serum, Seq-3, Seq-6, Seq-19 and Seq-54, were developed using whole-serum subtractive SELEX technology with *K*_d_ of 128 ± 26.3 nM, 149 ± 23.6 nM, 232 ± 44.2 nM, 202 ± 25.6 nM, respectively. These generated aptamers showed higher specificities toward their target serum by differentiating normal serum but closely related other cancer serums. The selected four high affinity DNA aptamers were further applied to the development based on qPCR method for the early detection of gastric cancer. In addition, we performed MALDI-TOF MS followed by secondary peptide sequencing MS analysis for the identification of the aptamer binding proteins. Among these potential biomarkers, APOA1, APOA4, PARD3, Importin subunit alpha-1 showed a relatively high score probability. Therefore, these four ssDNA aptamers generated in our study could be a promising molecular probe for gastric cancer diagnosis.

## Abstract 2

Gastric cancer is still among the leading causes of cancer deaths worldwide.

## Graphical abstract

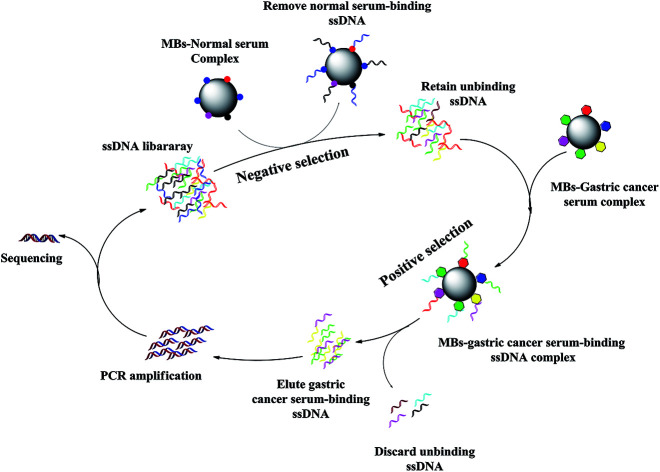

## Introduction

Gastric cancer is the second most common malignancy in the world, threatening seriously the human health.^1^ Therefore, early screening is the most critical for gastric cancer. Currently, Carcinoembryonic antigen (CEA) and CA125 are the commonly used tumor biomarkers in clinical practice for gastric cancer screening; early diagnosis, targeted treatment and prognosis are the main strategies to promote the five-year survival rate.^2–5^ However, the clinical diagnostic accuracy of CEA is unsatisfactory due to low sensitivity and specificity. Another major challenge for gastric cancer is the lack of therapeutic options. In addition, the 5 year survival rate of gastric cancer patients is very low with only about 5–10%.^6–8^ At present, the effective gastric cancer tumor biomarker get poor in clinical diagnosis and prognosis. Therefore, there is an urgent need to identify the factors responsible for gastric cancer in its early diagnosis.

Aptamers are a class of high-affinity nucleic acid ligands, which are selected through ssDNA or RNA binding a specific target molecule from a region library *in vitro*.^9,10^ The *in vitro* selection of an aptamer is referred to as the Systematic Evolution of Ligands by EXpotential enrichment(SELEX),^11,12^ which makes aptamer identification a tuneable and controllable process. Aptamers have been regarded as an alternative to antibodies. Aptamers display a few advantages over antibodies in biosensing and assays and can overcome some limitations of antibodies in analytical applications.^13,14^ Furthermore, aptamers are stable, non-toxic, lack immunogenicity and have rapid tissue penetration,^15–18^ making them very attractive tools for biomedical applications. In addition, aptamers can be easily modified with different function for labeling.^19–21^ Therefore, aptamer-based methods have emerged as attractive tools in biosensors; bioanalysis can be used for clinical detection and treatment to achieve early diagnosis and targeted therapy. Li had successfully screened to lung cancer serum aptamers using SELEX technology.^22^

Herein, we developed a whole-serum subtractive SELEX strategy for identifying aptamers that distinguish from other cancer serum samples. Gastric cancer serum is used as the target serum, which presents high gastric cancer potential. The normal serum is used as control serum for counter selection. After 10 cycles of selection, four ssDNA aptamers, Seq-3, Seq-6, Seq-19 and Seq-54, were able to specifically recognize gastric cancer serum. The selected aptamers were subjected to ELONA and qPCR to evaluate their binding affinity and selectivity. In addition, we performed MALDI-TOF MS followed by secondary peptide sequencing MS analysis for the identification of the aptamer binds proteins. Among these potential biomarkers, APOA1, APOA4, PARD3, Importin subunit alpha-1 showed a relatively high score probability. Therefore, these four DNA aptamers generated here could be a promising molecular probe for gastric cancer diagnosis.

## Materials and methods

### Random DNA library, primers, reagents and serum

A 88-nucleotide (nt) ssDNA library containing a 40 nt central random region flanked by two primer binding sites: 5′-CTATAGCAATGGTACGGTACTTCC-N_40_-CAAAAGTGCACGCTACTTTGCTAA-3′. The primers used for qPCR amplification were: forward, 5′-CTATAGCAATGGTACGGTACTTCC-3′ and reverse, 5′-Biotin-5′-TTAGCAAAGTAGCGTGCACTTTTG-3. Both the library and the primers were synthesized by Sangon Biotech (Shanghai, China). Carboxylated magnetic beads were provided by Thermo Fisher Scientific (USA).

The binding buffer solution was a mixture of 1 L of phosphate-buffered saline (1× PBS, Invitrogen Co., USA). A washing buffer solution containing 1 L of 1× PBS, 1 mL of 1.5 mmol MgCl_2_ and the Tris phosphate-buffered saline (TPBS) solution was a mixture of 1 L of 1× PBS with containing 1 mL of 1% (v/v) Tween-20, were stored at 4 °C. All related PCR reagents were purchased from Sangon Biotech (Shanghai, China).

The gastric cancer serum (Collecting 100 cases of early gastric cancer with pathological diagnosis), normal serum, lung cancer serum, colorectal cancer serum and hepatocellular carcinoma serum were supplied by the first hospital of Qinhuangdao, Hebei Province in China.

### *In vitro* selection of aptamers (SELEX)

In the process of SELEX, a scheme for the preparation of carboxylated magnetic beads (MBs) and the capturing of gastric cancer serum. After magnetic separation using the magnetic separator, 1 mL of MBs was washed three times with 1 mL of binding buffer. The beads were then mixed with 2 mL of gastric cancer serum and were incubated using a rotating mixer for 2 h at 37 °C. After magnetic separation, the MBs-serum complexes were washed three times with 500 μL of binding buffer and the complexes were subsequently collected by magnetic separation.

The initial ssDNA library (OD:1.0) was dissolved in 3 mL of binding buffer solution, and then were denatured by heating at 95 °C for 10 min before being cooled on ice for 10 min the MBs-serum complexes were washed three times with binding buffer and were followed by incubating with the initial ssDNA library for 1 h at 37 °C. The supernatant solution was discarded, and then the MBs-serum-ssDNA complexes were transferred to 200 μL of ddH_2_O in 1.5 mL EP tube, the MBs-serum-ssDNA complexes were washed three times with 3 mL of binding buffer. To elute the bound ssDNA, the MBs-serum-ssDNA complexes were heated at 95 °C for 10 min. After magnetic separation, the supernatant was collected and was used as a template for amplifying by qPCR using forward primer and biotin-labeled reverse primer under the following conditions: pre-denaturing at 95 °C for 7 min, followed by 20 cycles or 25 cycles of denaturing at 95 °C for 30 s, annealing at 60 °C for 34 s and extending at 72 °C for 1 min. After isolation by streptavidin-coated beads, 1 mL of 1 wt% Tris phosphate-buffered saline (TPBS) solution was added, and the mixture was incubated for 10 min at 40 °C. The ssDNA pool was collected and was used later for next round selection. The normal serum was used as the negative serum for subtractive selection. It was performed to maximize the removal of DNA sequences binding to normal serum. After normal serum incubation with evolved ssDNA pool, the supernatant was then collected and was applied on MBs-gastric cancer serum complexes for further positive selection. To acquire aptamers with high affinity and specificity, the incubation time with MBs-gastric cancer serum complexes was reduced from 60 min to 30 min as the number of selection cycles increased as well as the washing strength was increased by gradually increasing washing time (from 1 min to 3 min), washing volume (from 3 mL to 6 mL) and washing cycles (from 3 times to 5 times). After 10 rounds of selection, the evolved ssDNA pool was amplified by PCR.

### Next-generation sequencing

After ten selection cycles, the specifically enriched ssDNA library was PCR amplified with non-modified reverse primers, the PCR products were sent for high-throughput DNA sequencing analysis at the Sangon Biotech (Shanghai, China). Sequences were analyzed using NextGen Sequence Workbench v3.2.3. low quality reads and reads out of the range of 89–91 bases long were removed from the sequences pool. The most frequent and enriched sequences were chosen for further analysis. These candidate aptamers secondary structure was predicted by NUPACK.

### Binding affinity of candidate aptamers to gastric cancer serum by ELONA

The binding affinity of each individual aptamer for gastric cancer serum was assayed using an Enzyme Linked Oligonucleotide Assay (ELONA).^23,24^ In this assay the gastric cancer serum was adsorbed in the 1.5 mL of Eppendorf tube of MBs and was titrated with biotinylated aptamer, incubated and then the beads were washed with binding buffer. The amount of captured aptamer was then determined by adding streptavidin-HRP and suitable substrates and measuring the absorbance.^25–27^

200 μL of gastric cancer serum was added to each 1.5 mL of Eppendorf tube of 50 μL of MBs and was incubated at 37 °C overnight. After magnetic separation, the MBs were washed 3 times with 1 mL of TPBS (PBS supplemented with 1% (v/v) Tween-20) solution after being blocked with 1% (w/v) BSA. The biotin-labeled selected candidate aptamer was added to the individual gastric cancer serum coating MBs Eppendorf tubes at various quantities ranging from 250 nM to 4000 nM in the SELEX binding buffer, and then was incubated for 30 min at 37 °C. The unbound DNA was removed by washing 3 times with 1 mL of TPBS solution. The bound DNA was determined by adding 100 μL of a streptavidin-HRP solution and was incubated for 30 min at 37 °C and the unbound was discarded. The MBs were washed 3 times with 1 mL of the TPBS solution. The amount of HRP retained corresponded to the amount of bound biotinylated aptamer. It was done by measuring HRP activity using 100 μL of TMB substrate solution.^28^ The enzyme reaction was quenched by adding 100 μL of 2 M H_2_SO_4_. Finally, the absorbance at 450 nm was measured using a microplate reader (Bio-Tek).^29–31^ The obtained data were analyzed and then the *K*_d_ values of these candidate aptamers were calculated by non-linear regression analysis using OriginPro 9.0 software. The equation *Y* = *B*_max_*X*/(*K*_d_ + *X*) was used to calculate the median effective concentration.

### Specificity analysis

Specificities of four DNA aptamers were determined by using qPCR. For specificity experiments gastric cancer, lung cancer, colorectal cancer and hepatocellular carcinoma were used as target serum. 200 μL of gastric cancer serum was mixed with 100 μL of MBs for 30 min at 37 °C. After magnetic separation, the MBs were washed three times with binding buffer for 1 min each time, followed by incubation with the candidate aptamer for 30 min at 37 °C. After incubation, the supernatant solution was discarded, the MBs-serum-aptamers complexes were washed five times with the binding buffer for 1 min each time. Then, the MBs-serum-aptamers complexes were collected and were transferred to 100 μL of ddH_2_O. The serum-binding ssDNA was eluted by heating at 95 °C for 10 minutes. Finally, 6 μL of the supernatant containing the ssDNA was used to quantify through qPCR. Aptamer was amplified with Taq DNA polymerase using a SYBR® Green master mix. Samples were heated at 95 °C for 7 min followed by 40 cycles of denaturation at 95 °C for 30 s, annealing at 60 °C for 34 s, extension at 72 °C for 1 min and hold on 4 °C. The data were analyzed with the Rotor-Gene 6000 Series Software (Corbett Life Science, USA). All other experiment conditions were the same as described above. All of these serum were tested at 200 μL.

### Identification of candidate serological biomarkers

These four DNA aptamers (5 × 10^−6^ mol L^−1^, 20 μL) and 20 μL of gastric cancer serum were incubated respectively for 30 min at 37 °C normal serum as a negative control. Then, these complexes were separated on a 8% native polyacrylamide gel. The retarded band was excised and the protein components in it were recovered, digested with porcine trypsin and was subjected to MALDI-TOF MS followed by secondary peptide sequencing MS analysis and a Mascot database search for the identification of the mixture proteins.^32^ The corresponding position in the control lane (the complex of the initial ssDNA library and gastric cancer serum, the complex of these four DNA aptamers and normal serum) was excised and identified by MS simultaneously to exclude the protein mobilized similarly with the complex. The candidate serological biomarkers of gastric cancer were thus gained.

## Results and discussion

### Selection of ssDNA aptamers against gastric cancer serum

**Fig. 1:**
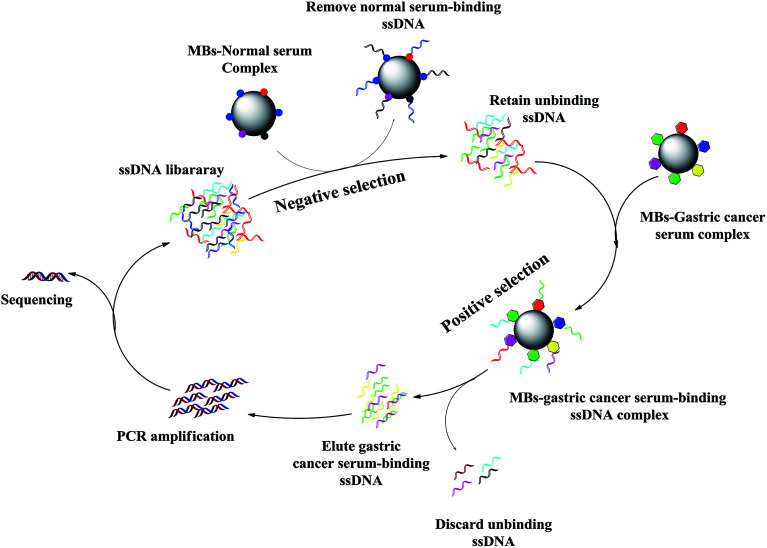
Schematic of the whole-serum subtractive SELEX process for gastric cancer serum. The DNA library was incubated with normal serum for negative control to remove nonspecific sequences. The unbound sequences were collected, and then incubated with gastric cancer serum. After washing, the bound sequences were eluted and amplified by PCR for next round selection. After 10 rounds of selection, the ssDNA library was successfully enriched, sequenced to identify individual aptamer sequences.

The strategy of the whole-serum subtractive SELEX in our work is illustrated in Fig. 1 with the detailed procedures. Compared with other partitioning methods, such as centrifugation, MBs possessed several advantages, including fast separation, ease of extensive washing in the SELEX. In this study, we used a library containing approximately 10^14^ (40 random nucleotides) different ssDNAs. Gastric cancer serum was used as target serum. Normal serum was used for counter selection. The normal serum was used as a negative serum to remove nonspecific sequences.

A total of ten rounds of aptamer screening were carried out, bounded DNA aptamers to target serum were isolated from aptamer pool of ssDNA library. Only a small amount of DNA remained after incubation, while the gastric cancer serum DNA sequences were amplified from each of the SELEX rounds. In order to select aptamers sensitively and specifically recognizing gastric cancer serum, each round of selection pressure was gradually enhanced (Table 1).

**Table 1:** Gastric cancer serum aptamers SELEX screening conditions

Round	MBs (mL)	ssDNA library (pmol)	ssDNA library into each round (pmol)	Gastric cancer serum (μL)	Library/MBs-serum	Incubation time (min)
1	4	69.5	69.5	600	2	60
2	4	46.3	46.3	300	2	60
3	3	79.8	52	350	4	50
4	3	59.7	50	300	4	50
5	2	42.9	40	275	6	50
6	2	51.6	45	250	6	40
7	2	49.1	40	225	8	40
8	1.5	30.1	30	200	8	40
9	1	28.1	28	175	10	30
10	1	31.0	29.6	150	10	30

**Fig. 2:**
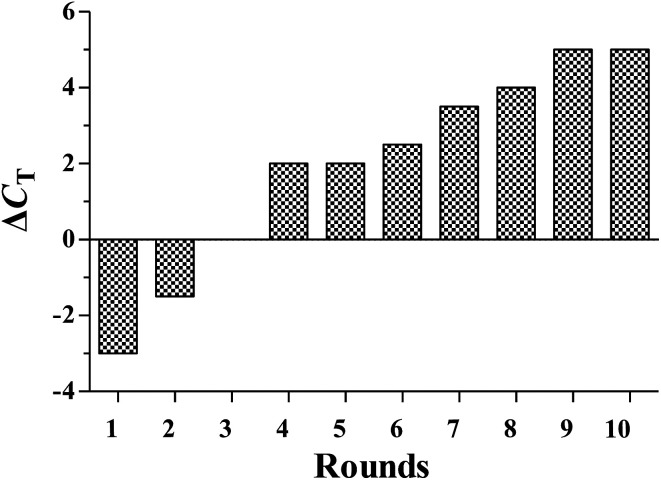
The binding affinity of the aptamer pools from the selected rounds was calculated using the qPCR method. Δ*C*_T_ = *C*_T_ (gastric cancer serum) − *C*_T_ (control normal serum).

During selection, the enrichment of ssDNA pools to the target serum was monitored by qPCR. The progression of the selection is represented by the Δ*C*_T_ value (Δ*C*_T_ = *C*_T_ (normal serum) − *C*_T_ (gastric cancer serum)). A Δ*C*_T_ value of more than 5 was observed between the selective target serum and negative control serum. In contrast, no change of Δ*C*_T_ value was observed on gastric cancer serum and control normal serum. These results indicated that the DNA sequences specifically bound to gastric cancer serum were enriched (Fig. 2). After sequencing, the aptamer candidates were grouped based on their sequential repeatability, secondary structures and homogeneity. Four representative sequences from different groups were chosen and chemically synthesized on a DNA synthesizer for further characterization (Table 2).

**Table 2:** Selected sequences found by NGS analysis and their frequency in the DNA library obtained after ten selection cycles

Name	Sequences	NGS(%)
Seq-2	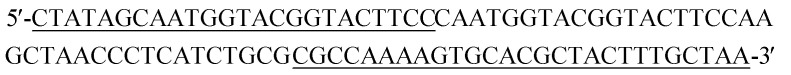	16%
Seq-3	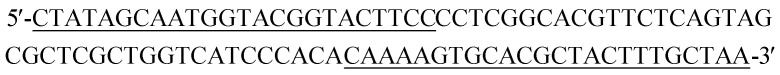	13%
Seq-6	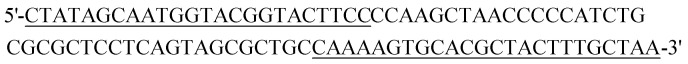	15%
Seq-19	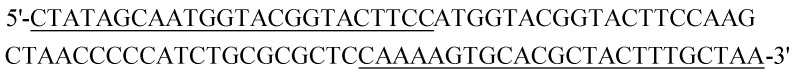	20%
Seq-25	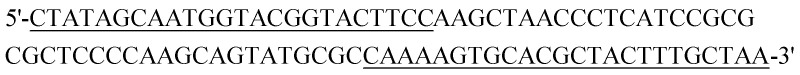	4%
Seq-32	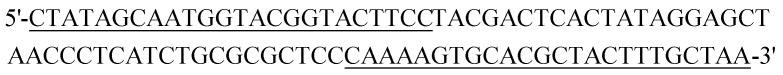	2%
Seq-51	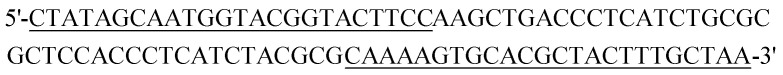	3.5%
Seq-54	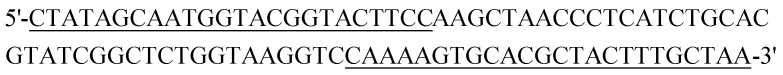	3%
Seq-69	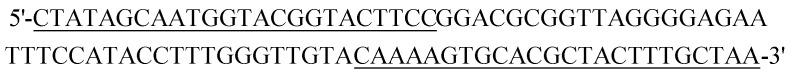	1.5%
Seq-76	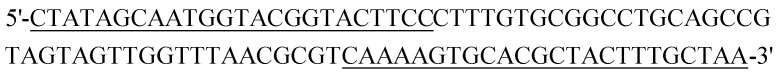	1%

### Characterization of aptamers

To identify individual aptamer candidates binding to gastric cancer serum, the highly enriched aptamer pool from the 10th round of the selection was sequenced using a high-throughput DNA sequencing device. Considering that the most frequently repeated sequence has been found to have the highest binding affinity. The binding affinities of these ten aptamers (Seq-2, Seq-3, Seq-6, Seq-19, Seq-25, Seq-32, Seq-51, Seq-54, Seq-69 and Seq-76) were assessed by measuring the dissociation constant (*K*_d_) using the ELONA assay. The results from ELONA demonstrated that aptamers Seq-3, Seq-6, Seq-19 and Seq-54 had good binding affinity to the gastric cancer serum but not to control normal serum. These results indicated that successful negative selection had been carried out and the selected aptamer Seq-3 may hold great potential as an excellent molecular probe for gastric cancer serum.

**Fig. 3:**
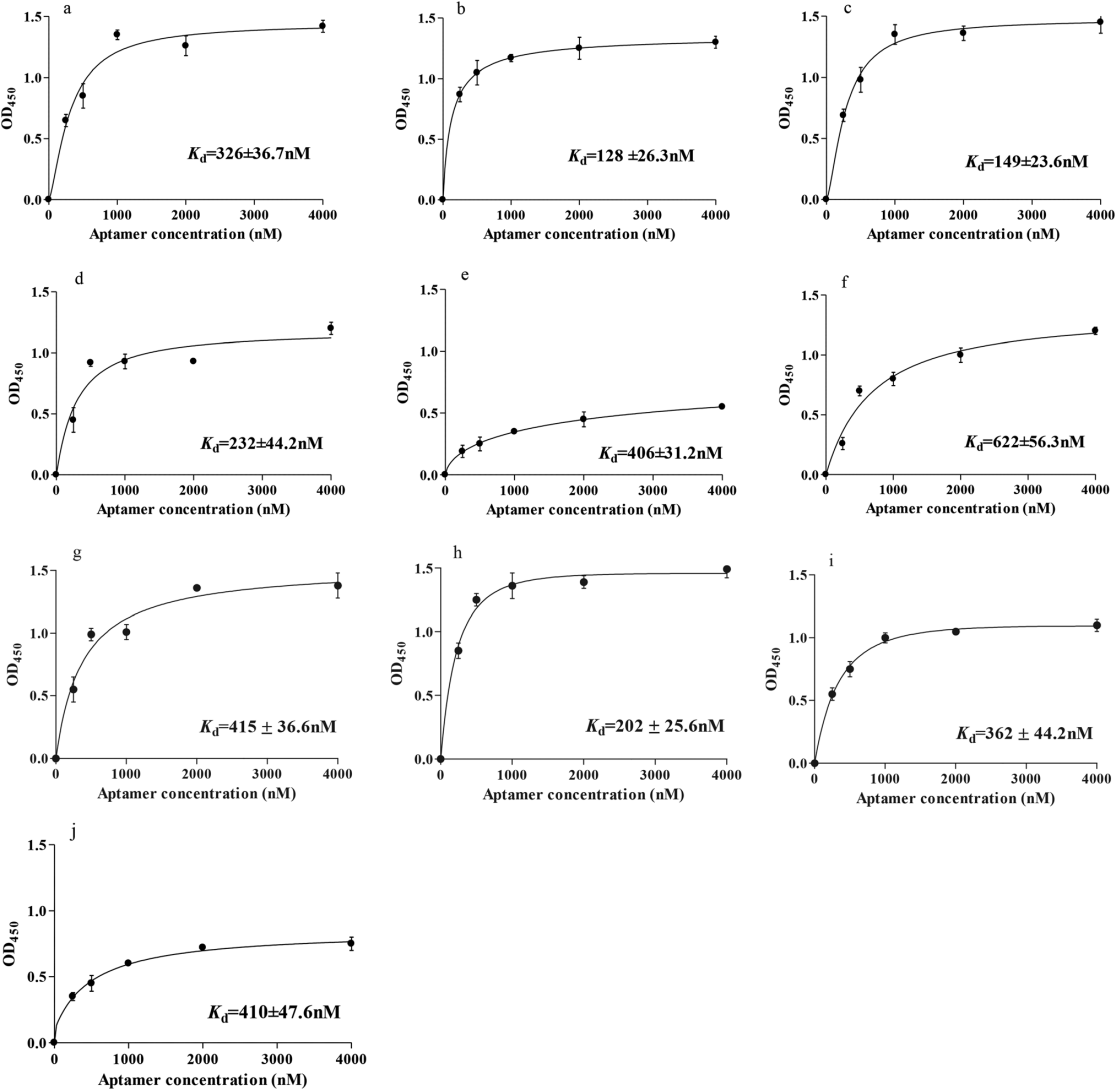
Binding saturation curve of the aptamers. (a–j): Representative Seq-2, Seq-3, Seq-6, Seq-19, Seq-25, Seq-32, Seq-51, Seq-54, Seq-69 and Seq-76 aptamer.

Subsequently, these aptamers were modified with biotinylated labeling. The resulting mean values were normalized and were plotted against the aptamer concentration with Sigma Blot software. The fitted *K*_d_ values were determined to be in the nanomolar level (326 ± 36.7 nM for Seq-2, 128 ± 26.3 nM for Seq-3, 149 ± 23.6 nM for Seq-6, 232 ± 44.2 nM for Seq-19, 406 ± 31.2 nM for Seq-25, 622 ± 56.3 nM for Seq-32, 415 ± 36.7 nM for Seq-51, 202 ± 25.6 nM for Seq-54, 362 ± 44.2 nM for Seq-69 and 410 ± 47.6 nM for Seq-76). Seq-3, these four DNA aptamers(Seq-3, Seq-6, Seq-19 and Seq-54) had the highest binding affinity with the lowest *K*_d_ value (Fig. 3).

**Fig. 4:**
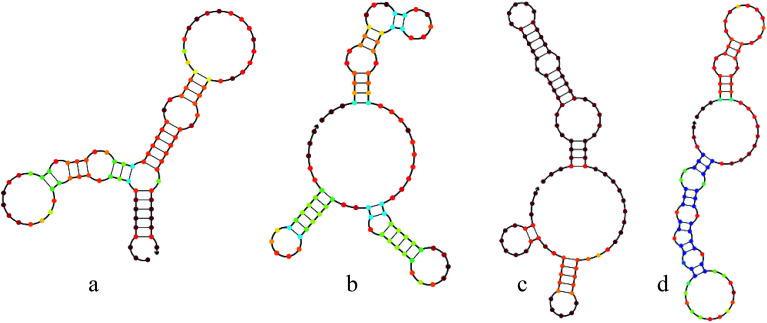
Structural analysis of these DNA aptamers at 37 °C were performed using NUPACK online tools to predict the secondary structure. (a–d): Representative Seq-3, Seq-6, Seq-19 and Seq-54 aptamer.

Aptamers can fold into complex and stable three-dimensional shapes, allowing them to bind to target molecules. Here, we also predicted the secondary structure of these aptamers by NUPACK and defined that three bulb-like stem-loop structures and a few unpaired nucleotides (Fig. 4).

### Specificity test

**Fig. 5:**
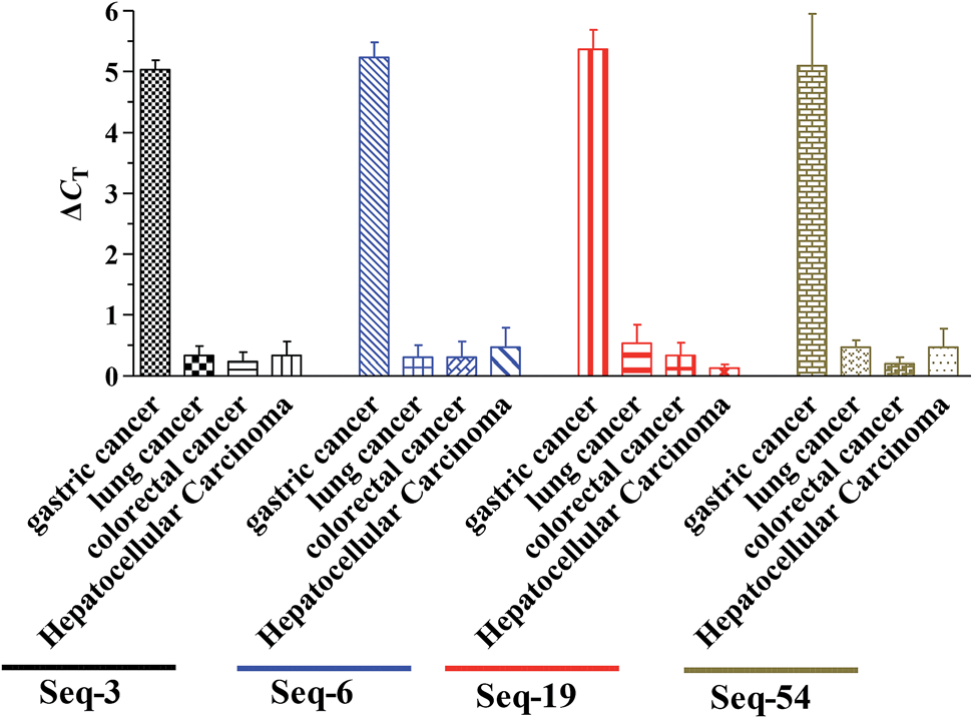
Specificity test of the aptamer-based assay using qPCR detection toward normal human serum sample, several different cancer serum samples lung cancer serum, colorectal cancer serum, hepatocellular carcinoma serum (each of 200 μL).

These four DNA aptamers (Seq-3, Seq-6, Seq-19 and Seq-54) were synthesized by Sangon Biotech (Shanghai, China). To further investigate the binding specificity of four DNA aptamers (Seq-3, Seq-6, Seq-19 and Seq-54), including 4 cancer serums (gastric cancer serum, lung cancer serum, colorectal cancer serum, hepatocellular carcinoma serum) and 1 normal serum, were separately incubated with four DNA aptamers and were tested by qPCR. The normal serum was used as negative control serum (Fig. 5). We used Δ*C*_T_ value to quantify the signals, as it directly relates to the relative difference in the nucleic acid template copy number between two samples. qPCR assay demonstrates specificity. For example, the Δ*C*_T_ value between the negative control and the few non-target serum (water replaces normal serum) was 0.5. In addition, a Δ*C*_T_ value of more than 5 was observed between the negative control and the non-target serum sample. Compared with the negative control signal, normal serum could cause a slight signal increase, but much lower than that from gastric cancer serum. This demonstrates that our obtained aptamers had a good selectivity for the discrimination of gastric cancer from other cancer serum.

We have demonstrated the capability to quantitatively detect serum samples using qPCR with DNA aptamers containing PCR primer sequences. By using an aptamer as an affinity and amplification reagent, the complex conjugation steps required in related methods such as immuno-PCR were avoided.

Four DNA aptamers were well applied to qPCR because of their small size (<100 nucleotides), and the capacity to modify sequences, which resulted in high efficiency amplification and increased assay sensitivity.

### Identification of candidate serological biomarkers

**Fig. 6:**
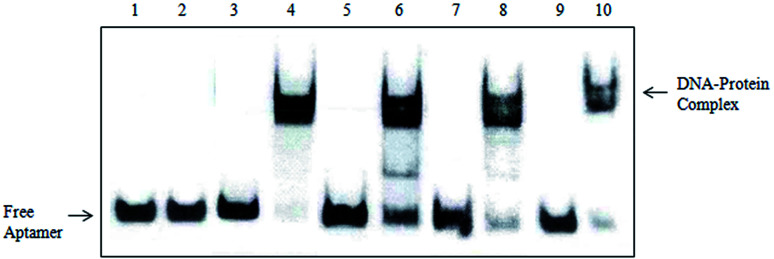
Electrophoretic mobility shift assay (EMSA) identification results (1): the complex of gastric cancer serum and initial ssDNA library, (2): the complex of normal serum and initial ssDNA library, (3): the complex of normal serum and Seq-3, (4): the complex of gastric cancer serum and Seq-3, (5): the complex of normal serum and Seq-6, (6): the complex of gastric cancer serum and Seq-6, (7): the complex of normal serum and Seq-19, (8): the complex of gastric cancer serum and Seq-19, (9): the complex of normal serum and Seq-54, (10): the complex of gastric cancer serum and Seq-54.

The slowly migrated band enriched the four DNA aptamers and gastric cancer serum complex in EMSA was excised and the protein components in it were recovered and were subjected to mass spectrometry analysis. The corresponding position in the control lane (the complex of the initial ssDNA library and gastric cancer serum, the complex of these four DNA aptamers and normal serum) was excised and identified by MS simultaneously to exclude the protein mobilized similarly with the complex. As shown in Fig. 6, the initial ssDNA library does not specifically bind to gastric cancer serum. However, these four DNA aptamers specifically recognized gastric cancer serum without binding to normal serum. We performed MALDI-TOF MS followed by secondary peptide sequencing MS analysis for the identification of the mixture proteins (Table 3). These Ig kappa chain C region, beta-2-glycoprotein 1, serum amyloid A-4 protein, Ig lambda chain V–III region also match in the control band. Potential tumor markers include APOA1, APOA4, PARD3, Importin subunit alpha-1. APOA1 had already been reported as a marker for the diagnosis of gastric cancer. It was shown to be up-regulated in gastric cancer patients by ELISA. In addition, APOA-I levels were significantly reduced after gastrectomy, indicating that the biomarkers reflect tumor burden.^33^ APOA4, PARD3, Importin subunit alpha-1 had already been reported elevated in other cancers.^34–36^ These four proteins showed a relatively high score probability. Moreover, further studies will focus on the relationships between the expressed levels of not reported potential biomarkers (APOA4, PARD3, Importin subunit alpha-1) and the diagnosis of gastric cancer.

**Table 3:** Potential biomarkers identified by MS

Aptamer	GI	Protein	Matched Peptides	Peptide
Seq-3	sp|P02647	APOA1	2	LLDNWDSVTSTFSK
				VSFLSALEEYTK
	sp|P01834	Ig kappa chain C region	1	KSGTASVVCLLNNFYPRE
Seq-6	sp|P50749	APOA4	1	MFLKAVVLTLALVAVAGARA
	sp|P02749	Beta-2-glycoprotein 1	1	MISPVLILFSSFLCHVAIA
Seq-19	Q8TEW0	PARD3	1	MKVTVCFGRTRVVVPCGDGH
	sp|P35542	Serum amyloid A-4 protein	1	MRLFTGIVFCSLVMGVTS
Seq-54	P52292	Importin subunit alpha-1	1	STNENANTPAARLHRFKNKGKDSTEMRRRR
	sp|P80748	Ig lambda chain V-III region	1	MAWTVLLLGLLSHCTGS

## Conclusions

Early detection of gastric cancer is an important process in preventing cancer disease. Naturally, not only early detection but also accurate identification is needed. For this purpose, aptamers might be an effective tool in the early diagnosis of tumor. In the present study, we successfully identified aptamers that specifically recognized gastric cancer *via* whole-serum subtractive SELEX and showed that these aptamers can be used in the detection using qPCR. Four highly specific aptamers generated for gastric cancer serum, Seq-3, Seq-6, Seq-19 and Seq-54, were developed using whole-serum subtractive SELEX technology with *K*_d_ of 128 ± 26.3 nM, 149 ± 23.6 nM, 232 ± 44.2 nM, 202 ± 25.6 nM, respectively. These generated aptamers showed higher specificities toward their target serum by differentiating normal serum but closely related other cancer serums. It will provide a novel way for the early diagnosis of gastric cancer. In addition, the fast response time of 1 h demonstrated the potential of the selected aptamer to be incorporated into various formats of aptamer biosensors for the rapid detection and enrichment of gastric cancer serum outbreaks.

We have demonstrated, for the first time, the effective use of aptamer amplification for a sensitive detection of serum samples. The combined approach of MBs separation, sandwich ELISA, and qPCR amplification of aptamers could be used to discriminate gastric cancer from other cancer serums and the signal represented by the Δ*C*_T_ values of qPCR. Conclusively, these results presented in this study confirm the possibility of aptamers as a useful tool to detect and diagnosis gastric cancer. We also performed MALDI-TOF MS followed by secondary peptide sequencing MS analysis for the identification of the aptamer binds proteins. Among these potential biomarkers, APOA1, APOA4, PARD3, Importin subunit alpha-1 showed a relatively high score probability. Therefore, these four ssDNA aptamers generated here could be a promising molecular probe for gastric cancer diagnosis.

## Conflicts of interest

There are no conflicts to declare.

## Supplementary Material
